# Hybrid El Tor *Vibrio cholerae* O1, Kuwait

**DOI:** 10.3201/eid1511.090357

**Published:** 2009-11

**Authors:** Rajinder M. Joshi, M. John Albert

**Affiliations:** Yiaco Medical Company, Ahmadi, Kuwait (R.M. Joshi); Kuwait University Faculty of Medicine, Jabriya, Kuwait (M.J. Albert)

**Keywords:** Cholera, Vibrio cholerae O1, hybrid variant, bacteria, letter, Kuwait, enteric infections

**To the Editor:** The traditional causative agent of cholera, *Vibrio cholerae* O1, has 2 biotypes, classical and El Tor. The current seventh pandemic that began in 1961 and has spread to much of the world is caused by the El Tor biotype. This biotype has replaced the classical biotype responsible for the previous pandemics. The classical and El Tor biotypes are differentiated by phenotypic tests (*1*), and several nucleotide base differences occur at positions 115 and 203 in the *ctxB* gene (C in both positions in the classical and T in both positions in the El Tor biotype). These differences translate to histidine at amino acid position 39 and threonine at amino acid position 68 for the B subunit of cholera toxin (CT) in the classical biotype and tyrosine and isoleucine, respectively, for the corresponding amino acids in the El Tor biotype (*2*).

Recently, 3 variants of the El Tor biotype have been found. These are the Matlab variants, which could not be biotyped because they have a mixture of classical and El Tor traits (*1*); the Mozambique variant, which has a typical El Tor genome but a tandem repeat of the classical CTX prophage located in the small chromosome (*3*); and the hybrid El Tor variant, which has a typical El Tor biotype and an El Tor CTX prophage but produces CT of the classical type (*4*).

This hybrid El Tor variant has replaced the El Tor biotype in Dhaka and other parts of Bangladesh (*4*) and in India (*5*), Japan, Hong Kong, Zambia, the People’s Republic of China, Sri Lanka, and Vietnam (*6*). Kuwait was affected by cholera in the mid 1960s during the current seventh pandemic. Subsequently, cholera disappeared from Kuwait as living standards improved. Screening of ≈5,000 acute-phase diarrheal stool samples in the mid-1980s in a major hospital in Kuwait did not yield *V. cholerae* O1 (*7*). The occasional cholera cases detected in Kuwait are imported, mainly from Asia through expatriate workers.

Two adult men, one who had just arrived from India and one who had just arrived from the Philippines, were admitted with severe watery diarrhea, vomiting, and dehydration to the Al-Adan Hospital, Kuwait, in November and December 2008, respectively. The patient from India reported eating in restaurants, and the patient from the Philippines had consumed fish soup just before the journey. Both patients were initially rehydrated with intravenous fluids.

Stool cultures were performed by using a battery of media, including thiosulfate citrate bile salt sucrose (TCBS) agar. Yellow colonies from TCBS agar were tested by using MicroScan WalkAway 96 (Dade Behring, West Sacramento, CA, USA) panel NBPC 34 and API 20E strip (bioMérieux, Marcy l’Etoile, France), which suggested *V. cholerae*. In slide agglutination tests, the colonies agglutinated with *V. cholerae* O1 polyvalent antiserum and Ogawa serotype antiserum (Denka-Seiken, Tokyo, Japan). The isolate from the patient from India was susceptible to tetracycline and ampicillin and resistant to co-trimoxazole, but the isolate from the patient from the Philippines was susceptible to all 3 of these antimicrobial agents in MicroScan (Dade Behring) and disk diffusion tests. Both patients were successfully treated with rehydration therapy and intravenous vibramycin (a semisynthetic tetracycline). Both *V. cholerae* O1 Ogawa isolates showed positive results in Vogues-Proskauer, chicken cell agglutination, and tube hemolysin tests and were resistant to polymyxin B (50 international units), results that suggest the El Tor biotype (*1*).

Both isolates were positive for the *ctxA* gene and El Tor–specific *tcp* gene but negative for classical-specific *tcp* gene by PCR (*8*). The genotype of the *ctxB* gene was determined by a mismatch amplification mutation assay PCR that detects polymorphism at nucleotide position 203; both isolates yielded a 186-bp amplicon with the classical biotype-specific primers and no amplicon with the El Tor biotype-specific primers (*9*).

The classical *ctxB* genotype of the isolates was further confirmed by sequencing the *ctxB* gene by using the BigDye termination method (Applied Biosystems, Foster City, CA, USA) with specific primers (*10*). This sequencing showed that both isolates had histidine at position 39 and threonine at position 68 in CT-B subunit. Thus, the isolates are hybrid variants that are phenotypically El Tor but genotypically classical for the *ctxB* gene.

Our findings suggest that cholera caused by the hybrid variant is present in the Philippines. The 2 cholera cases reported here were imported into Kuwait by travelers from cholera-endemic regions and were not endemic illnesses. The hybrid variant could possibly initiate the next cholera pandemic. Also, because CT is related to the major clinical sign of the disease, genetic changes in the molecule could result in alteration in the manifestation of the disease. The changes could also influence the effectiveness of cholera vaccines that contain CT-B as a component (*6*).
